# Validation of the pathological narcissistic inventory (PNI) and its brief form (B-PNI) in the Arabic language

**DOI:** 10.1186/s12888-023-04644-9

**Published:** 2023-03-15

**Authors:** Diana Malaeb, A. Esin Asan, Feten Fekih-Romdhane, Vanessa Azzi, Abir Sarray El Dine, Souheil Hallit, Aaron L. Pincus

**Affiliations:** 1 College of Pharmacy, Gulf Medical University, Ajman, United Arab Emirates; 2 School of Pharmacy, Lebanese International University, Beirut, Lebanon; 3 Pennsylvania State University, University Park, PA USA; 4 Faculty of Medicine of Tunis, Tunis El Manar University, Tunis, Tunisia; 5 The Tunisian Center of Early Intervention in Psychosis, Department of Psychiatry Ibn Omrane, Razi Hospital, Tunis, Tunisia; 6 School of Medicine and Medical Sciences, Holy Spirit University of Kaslik, P.O Box 446, Jounieh, Lebanon; 7 Department of Biomedical Sciences, School of arts and Sciences, Lebanese International University, Beirut, Lebanon; 8 Research Department, Psychiatric Hospital of the Cross, Jal Eddib, Lebanon; 9 Applied Science Research Center, Applied Science Private University, Amman, Jordan

**Keywords:** Pathological narcissistic inventory, Psychometric properties, Arabic language, PNI, B-PNI

## Abstract

**Background:**

The Pathological Narcissism Inventory (PNI) is a multidimensional measure developed to assess narcissistic grandiosity and narcissistic vulnerability. We aimed to validate the Arabic version of the original Pathological Narcissistic Inventory (PNI) and its brief form (B-PNI) in a community sample of Lebanese adults.

**Methods:**

The English language PNI items were translated into Arabic following a rigorous translation, back-translation, and linguistic evaluation. A total of 401 participants were administered the translated PNI, as well as previously validated Arabic versions of the Big Five Inventory-2, the Rosenberg Self-esteem Scale, the Patient Health Questionnaire (PHQ-9), and the Impulsivity-8 Scale.

**Results:**

Exploratory and confirmatory analyses supported the existence of seven first-order and two second-order factors of the PNI and B-PNI. Except for exploitativeness where females scored lower than males, no other significant differences by gender were observed for the remaining PNI subscale scores. Additionally, scores on all the subscales exhibited good reliability, while the associations with external measures supported the concurrent validity of the translated instrument.

**Conclusion:**

The results of this study suggest that scores on the PNI and B-PNI are highly reliable with satisfactory concurrent and factorial validity, providing an assessment of broadly defined pathological narcissism among the Lebanese young adult population. The availability of the Arabic PNI and its brief form should facilitate improved understanding of pathological narcissism in Arabic cultures and the different factors that govern narcissistic personality pathology.

## Background

Narcissistic personality pathology is manifested by the two core themes of narcissistic vulnerability and narcissistic grandiosity [1]. Grandiosity is conceptualized as an exaggerated drive for self-enhancement, a sense of uniqueness and superiority, interpersonal exploitativeness, entitled expectations for self and others, fantasies of unlimited success, a tendency to repress negative aspects of the self, and a lack of empathy [1, 2]. Vulnerability is characterized by a fragile self-concept prone to deep shame regarding expectations, needs, and threats to self-esteem [3, 4]. Both vulnerability and grandiosity share features of entitlement and antagonism, and mirror the general aim of preserving a positive self-image through self-enhancement, admiration-seeking, and defensive strategies [2, 4, 5]. Narcissism is a complex and crucial phenomenon that has attracted the interest of the scientific community in the fields of clinical psychiatry and psychology for several decades now [1, 2]. However, a consensual definition of the narcissism construct is still lacking, especially given that the narcissistic personality disorder (NPD) diagnostic criteria do not reflect the well-known complexity of this personality’s characteristics. Particularly, the major gap in the diagnosis is the insufficient coverage of vulnerable criteria, which are similarly absent in instruments developed to assess NPD [1]. Therefore, recent efforts have been directed towards developing novel measures to resolve this issue and develop more comprehensive measures of the narcissism construct [6].

To better reflect the wide differences in the phenotypic presentation of pathological narcissism, Pincus et al. developed the Pathological Narcissism Inventory (PNI), which assesses self-reported narcissistic vulnerability and narcissistic grandiosity [7]. The PNI became then one of the most widely used research instruments of narcissism. It includes seven subscales serving as markers for intercorrelated higher order factors detecting the two aspects of pathological narcissism (vulnerability and grandiosity) [8]. Nevertheless, this 52-item form may be burdensome to administer in some settings or situations due to its length. Recently, a shorter version of the PNI has been validated, i.e. the Brief-Pathological Narcissism Inventory (B-PNI) [9]. In this version, the 28 best-performing items have been selected from the original PNI, based on item response theory analyses. Thus, the B-PNI offers potential advantages, as it requires a shorter administration time and reduces respondents’ burden. It also provides a psychometrically cleaner version of PNI, and delivers a formulation that better differentiates between vulnerability and grandiosity [10]. The PNI does not assess a disorder, but rather it was constructed as a general multidimensional measure of pathological narcissism for the general population, and it performs well with such respondents [11]. Further the PNI and many of its translations were developed in university students and community dwelling adult samples (e.g., [7, 12, 13]. When the PNI structure and psychometrics are examined in clinical samples, it is found to be psychometrically consistent with non-clinical samples (e.g. [14, 15]).

Evaluating narcissism has demonstrated clinical utility and validity. Indeed, previous studies find that PNI scores are associated with both pathological and normal personality traits. Regarding the former, both PNI vulnerability and PNI grandiosity are negatively associated with Agreeableness, but they diverge with other Five-factor Model domains. Specifically, PNI grandiosity is negatively related to Neuroticism and positively associated with Extraversion. In contrast, PNI vulnerability is negatively associated with Extraversion, Conscientiousness, and Openness, and strongly positively related with Neuroticism. These patterns are consistent with the trifurcated trait model of narcissism that includes self-centered antagonism (i.e., low agreeableness), narcissistic neuroticism, and agentic extraversion [16, 17]. Regarding the latter, PNI grandiosity exhibits positive associations with several externalizing traits such as manipulativeness, deceitfulness, attention-seeking, and risk-taking. PNI vulnerability exhibits unique positive associations with other distinct externalizing traits (e.g., hostility, irresponsibility, callousness) while also exhibiting broader associations with other pathological trait domains (e.g., intimacy avoidance, anhedonia, depressivity, eccentricity). Overall, this pattern is consistent with the results of a large literature that finds PNI grandiosity and PNI vulnerability share an antagonistic core while aligning PNI grandiosity with externalizing traits and symptoms, and PNI vulnerability with both internalizing and externalizing traits and symptoms [18].

Consistent with associations found for internalizing and externalizing traits, problems, and psychopathology, PNI vulnerability and PNI grandiosity exhibit distinct links with core affect, self-conscious emotions, and self-esteem. PNI Vulnerability is negatively related with self-esteem, whereas PNI grandiosity is positively correlated with self-esteem [7]. Additionally, PNI vulnerability is independently associated with daily fluctuations in self-worth feelings [19]. PNI vulnerability is inversely correlated with authentic pride, positively correlated with hubris and shame, and unrelated to guilt. In contrast, PNI grandiosity is positively associated with guilt and unrelated to pride and shame [7]. PNI vulnerability is inversely correlated with positive affectivity, and positively correlated with envy, rage, and negative affectivity; while PNI grandiosity only showed positive relationship with positive affectivity [14].

Narcissistic vulnerability and grandiosity are also linked to interpersonal difficulties. PNI grandiosity is related to intrusive problematic behaviors, domineering, and predominately vindictive[7]. Similarly, PNI vulnerability is related to avoidant and exploitable problems, as well as vindictive interpersonal problems [7]. Narcissistic vulnerability and grandiosity also show differential correlations with the utilization of psychotherapy and psychiatric treatment. For instance, Ellison and colleagues [20] showed that narcissistic grandiosity was inversely related to treatment utilization (inpatient admissions, partial hospitalizations, telephone-based crisis services, taking medications) and positively related to outpatient therapy no-shows. Narcissistic vulnerability was positively linked to inpatient admissions, outpatient therapy sessions (both cancelled and attended), and the use of telephone-based crisis services. Results indicating that narcissistic vulnerability shows positive associations with treatment utilization support prior evidence that patients with narcissistic traits possibly present for mental health services when their vulnerable self-state becomes predominant [21]. In addition, PNI vulnerability and PNI grandiosity are both associated with suicidal ideation and suicide attempts [7, 22, 23], and non-suicidal self-injury [24].

Several studies have examined the associations of global pathological narcissism using the PNI total score. A study by Kealy et al. [11] found that after controlling for general psychiatric distress, the PNI total score predicted substance use, aggressiveness, anger, and risk-taking behaviors in a national sample of Canadian men. In a prospective study of depressive symptoms [25], PNI scores were positively linked to mean level, variability, and instability of depression levels over 8 weeks. The pattern of results suggests that pathological narcissism is associated with episodic, rather than chronic, depressive symptoms, particularly anhedonia. Results of a study examining pathological narcissism and exposure to war (missile attacks toward Israel) demonstrated a significant relationship between acute anxiety symptoms and exposure severity for people with high pathological narcissism levels (greater PNI scores) but not for those with low pathological narcissism levels [26]. Therefore, subjects with pathological narcissism features seem to be at a heightened risk for the development of acute anxiety after exposure to a life-threatening event. Finally, a study examining the effect of pathological narcissism on caregiver burden [27] found that caregivers of highly narcissistic individuals endorsed greater burden than caregivers of individuals with other mental diseases. Caregivers of persons high in pathological narcissism also displayed low levels of well-being similar to that of people diagnosed with mood, depressive, and anxiety disorders.

Validated translations of the PNI are available in several languages including Turkish [28], German [14], Chinese [29], Japanese [30], Italian [15], and French [12]. However, no Arabic version of the scale is available to date. In addition, despite research indicating that narcissism is shaped by cultural characteristics and sociocultural changes [31, 32], we could find only one previous study assessing this construct in Arab people [33]. It found that Arab female students (from the United Arab Emirates) exhibited greater narcissism than their western counterparts (from the UK), contradicting the initial assumptions that narcissism would be higher in people from individualist and developed countries. Therefore, cultural differences in narcissism are still unclear and need further research. For this, we believe that the first step toward expanding our knowledge on cross-cultural variations of narcissism is to develop translated versions of the PNI; especially in under-researched countries. In addition, whenever a short scale is preferred over its longer original version which measures the same construct, a compromise between resource savings and the potential loss of psychometric quality certainly arises [34]. Thus, the current study sought to examine the psychometric characteristics of an Arabic translation of the original Pathological Narcissistic Inventory (PNI) and its brief form (B-PNI) in a non-clinical sample of Arabic-speaking community adults from Lebanon. We hypothesized that the PNI and B-PNI will show seven factors (H1) and each will have an adequate internal consistency (H2). Further, we expected that the PNI and B-PNI total, grandiosity, and vulnerability scales will show patterns of associations with personality traits, self-esteem and depression constructs in the expected directions, providing evidence of validity of scores on the instruments. Specifically, we hypothesized, in light of the available literature [35–37], that grandiosity will be correlated with more adaptive personality traits, higher self-esteem, more impulsiveness, and less depression; whereas vulnerability will show positive correlations with maladaptive personality traits, depression, and negative correlations with self-esteem and impulsiveness.

## Methods

### Participants

Young adults (N = 401; 71.8% females) from all governorates of Lebanon were invited to complete the survey. Participants had a mean age of 22.48 years (SD = 3.58), ranging from 18 to 30 years. Most participants were single (85.5%) and had a university level of education (83.8%).

### Study design

One certified interpreter translated the PNI scale from English into Arabic, while a second interpreter translated the Arabic version back into English. Then, the two English versions were assessed by a committee consisting of the research team and the two translators. There were very minor discrepancies in the translation of both scales, such as word choice differences, which were resolved through consensus. All items of the Arabic PNI are presented in Appendix 1.

Data was collected between January and May 2022, through the snowball technique. The research team initiated the first contact with some participants by sending them the Google form link to the questionnaire; those participants were solicited to diffuse the link to other potential participants. The link contained a brief introduction related to information about the study (e.g., objectives of the study, confidentiality of answers, estimated duration, etc.). Those who agreed to participate answered “yes” to the question related to their full willingness to be part of the study. The questionnaire took approximately 30 min to complete. Participation was voluntary, with no remuneration was given to any participant in return.

### Measures

#### Sociodemographic information

We collected information about age, gender, marital status and education level.

#### Pathological Narcissistic Inventory

This scale is a 52–item 6-point scale that ranges from 0 (*Not at all like me*) to 5 (*Very much like me*) [7].Three subscales assess facets of grandiosity (grandiose fantasy—GF, self-sacrificing self-enhancement–SSSE, exploitativeness–EXP) and four subscales assess vulnerability (hiding the self—HS, devaluing–DEV, entitlement rage–ER, contingent self-esteem–CSE). The B-PNI assesses the same 7 subscales using 4 items each (28 items total).

#### Big Five Inventory-2

Validated in Arabic [38], this is a 60-item five-point Likert scale, rated from 1 (strongly disagree) to 5 (strongly agree). Five personality traits are obtained from this scale as follows: open-mindedness (α = 0.51), agreeableness (α = 0.66), negative emotionality (α = 0.64), extroversion (α = 0.57), and conscientiousness (α = 0.76).

#### Personality Inventory for DSM-5—Brief Form (PID-5-BF)

Validated in Arabic [39], this scale is composed of 25 items, rated on a scale from 0 (very false or often false) to 3 (very true or often true) [35]. Five scores derive from this scale as follows: negative affect (α = 0.74), detachment (α = 0.72), antagonism (α = 0.74), disinhibition (α = 0.70) and psychoticism (α = 0.78). Higher scores indicate greater personality dysfunction in each domain.

#### Patient Health Questionnaire (PHQ-9)

Validated in Lebanon [40], this scale contains 9 items that evaluate the severity of depression. Higher scores reflect more severe depressive symptoms (α = 0.87).

#### Rosenberg self-esteem scale

Validated in Arabic [41], this scale contains 10 items, scored on a four-point Likert scale, ranging from 1 (strongly disagree) to 4 (strongly agree). Higher total scores indicate greater self-esteem levels (α = 0.79).

#### Impulsivity-8 scale

This is a 6-point scale ranging from 0 (doesn’t apply at all) to 5 (applies completely). Greater scores reflect more impulsivity in each of the four subscales: perseverance (αCronbach = 0.65) premeditation (α = 0.79), urgency (α = 0.67), and sensation seeking (α = 0.68). The Arabic validated version used in the present study confirmed the original factor structure of the scale [42].

### Statistical analyses

#### Factor analyses

We conducted item-level confirmatory factor analyses (CFA) to verify both the first-order and the second-order factor structures of the Arabic PNI and B-PNI to determine whether they replicate the hierarchical structure of the original scales [8]. All factor analyses were performed using the lavaan package in R version 4.1.1. Our models for both the Arabic PNI and B-PNI consisted of two correlated higher-order factors of Narcissistic Vulnerability (NV) and Narcissistic Grandiosity (NG) and the seven lower-order factors. Consistent with the factor structure confirmed by Wright and colleagues [8], we allowed EXP, SSSE and GF to freely load onto NG, and HS, CSE, DEV, and ER to freely load onto NV. Due to the rank ordinal nature of the 6-point Likert scale, we opted to use the weighted least squares mean and variance adjusted (WLSMV) estimator for our CFA. The WLSMV estimator is generally the least biased and most accurate estimator for rank ordinal data and has been shown to work well if sample size is 200 or larger [43, 44].

Although the chi-square (χ2) is the most commonly used summary statistic to examine model fit, it possibly overestimates lack of fit with large samples and a large number of model parameters [45]. Therefore, we opted to use the root mean square error of approximation (RMSEA), the Tucker-Lewis index (TLI), the comparative fit index (CFI), and the standardized root mean square (SRMR) to evaluate model fit. We accepted model fit if three out of four of the fit indices met the following cut-offs: RMSEA < 0.08, TLI > 0.95, SRMR < 0.08, CFI > 0.95 [46].

All PNI subscales scores were considered normally distributed as evidenced by skewness and kurtosis values varying between − 1 and + 1 [47]. Pearson test was used to correlate continuous variables between each other, whereas the Student t test was used to compare scores between sexes. Consistent with the approach of Edershile and colleagues [48], linear regression analyses for each external validity variable were run twice. In Model 1, grandiosity and vulnerability were individually entered as separate independent variables, and in Model 2, grandiosity and vulnerability were both entered concurrently as independent variables. This helped determine the unique contribution of each scale as reflected in R^2^ changes from the two univariate models, similar to the method used by Edershile and colleagues [48]. *P* < .05 was deemed statistically significant.

## Results

**Fig. 1:**
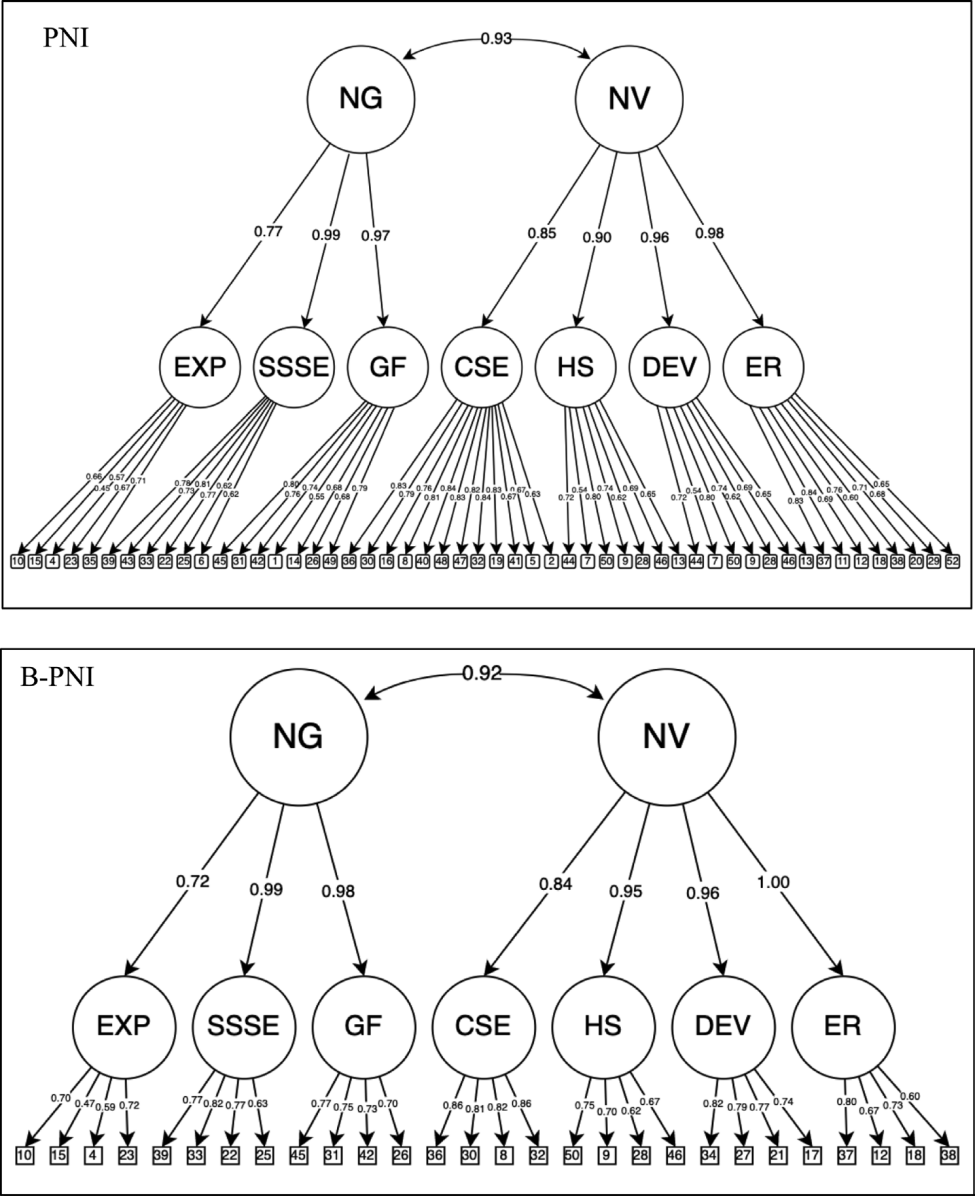
Hierarchical Structure of the Pathological Narcissism Inventory and Brief-Pathological Narcissism Inventory

Table 1 shows a summary of fit statistics for both CFA models. Due to the large sample size and large number of parameters, χ2 values were significant as expected. In our analyses, the CFI and TLI (both of which consider sample size and model complexity) are good, and the SRMR is adequate, but the RMSEA were near but above the cut-off. It is somewhat of an interesting fit profile, in that typically RMSEA would be more forgiving in this situation, which is a high df model. At the same time, the TLI is often very unforgiving with high df models (even when the CFI is favorable) and it indicates good fit. On balance here we would say this seems reasonable, especially given it is very hard to get good fitting models at all in CFA with a large number of items. Tables 2 and 3 summarize the standardized factor loadings of the Arabic versions of the original and brief PNI scales respectively. Figure 1 illustrate the hierarchical factor structures of the original and brief PNI scales.

**Table 1:** Summary of Fit Statistics for the Arabic PNI and B-PNI Hierarchical CFA Models

Model	χ^2^ (*df*)	p	CFI	TLI	RMSEA	SRMR
PNI	5381.60 (df = 1267)	< 0.001	0.979	0. 979	0.090	0.079
B-PNI	1328.65 (df = 342)	< 0.001	0.984	0.982	0.085	0.072

*Note*. PNI = Pathological Narcissism Inventory; df = degrees of freedom; χ^2^ = chi-square fit statistic; TLI = Tucker–Lewis index; CFI = comparative fit index; SRMR = standardized root mean square; RMSEA = root mean square error of approximation. *N* = 401

**Table 2:** Standardized factor loadings of the Arabic PNI items

PNI Item Numbers	PNI Lower-Order Factors						
	EXP	SSSE	GF	CSE	HS	DEV	ER
10	0.66						
15	0.45						
4	0.57						
23	0.67						
35	0.71						
39		0.78					
43		0.73					
33		0.81					
22		0.77					
25		0.62					
6		0.62					
45			0.80				
31			0.76				
42			0.74				
1			0.55				
14			0.68				
26			0.68				
49			0.79				
36				0.83			
30				0.79			
16				0.76			
8				0.81			
40				0.84			
48				0.83			
47				0.82			
32				0.84			
19				0.83			
41				0.67			
5				0.67			
2				0.63			
50					0.80		
9					0.74		
28					0.62		
46					0.69		
44					0.72		
7					0.54		
13					0.65		
34						0.81	
27						0.77	
21						0.77	
17						0.71	
24						0.64	
3						0.67	
51						0.76	
37							0.83
11							0.74
12							0.69
18							0.76
38							0.60
20							0.71
29							0.68
52							0.65
PNI Higher-Order Factors							
Narcissistic Grandiosity	0.77	0.99	0.97				
Narcissistic Vulnerability				0.85	0.90	0.96	0.98

*Note*. PNI = Pathological Narcissism Inventory; SSSE = Self-Sacrificing Self-Enhancement; HS = Hiding the Self; CSE = Contingent Self-Esteem; ER = Entitlement Rage; GF = Grandiose Fantasy; EXP = Exploitativeness; DEV = Devaluing;. *N* = 401

**Table 3:** Standardized factor loadings of the Arabic B-PNI items

Original PNI Item Numbers	B-PNI Lower-Order Factors						
	EXP	SSSE	GF	CSE	HS	DEV	ER
10	0.70						
15	0.47						
4	0.59						
23	0.72						
39		0.77					
33		0.82					
22		0.77					
25		0.63					
45			0.77				
31			0.75				
42			0.73				
26			0.70				
36				0.86			
30				0.81			
8				0.82			
32				0.86			
50					0.75		
9					0.70		
28					0.62		
46					0.67		
34						0.82	
27						0.79	
21						0.77	
17						0.74	
37							0.80
12							0.67
18							0.73
38							0.60
B-PNI Higher-Order Factors							
Narcissistic Grandiosity	0.72	0.99	0.98				
Narcissistic Vulnerability				0.84	0.95	0.96	1.00

*Note*. B-PNI = Brief Pathological Narcissism Inventory; CSE = Contingent Self-Esteem; EXP = Exploitativeness; SSSE = Self-Sacrificing Self-Enhancement; GF = Grandiose Fantasy; DEV = Devaluing; ER = Entitlement Rage; HS = Hiding the Self. *N* = 401

PNI = Pathological Narcissism Inventory; B-PNI = Brief Pathological Narcissism Inventory; NG = Narcissistic Grandiosity; NV = Narcissistic Vulnerability; CSE = Contingent Self-Esteem; EXP = Exploitativeness; SSSE = Self-Sacrificing Self-Enhancement; GF = Grandiose Fantasy; DEV = Devaluing; ER = Entitlement Rage; HS = Hiding the Self.

### Reliability

The PNI/B-PNI subscales yielded the following Cronbach’s alpha values: hiding the self (α = 0.83/0.81), contingent self-esteem (α = 0.93/0.59), self-sacrificing self-enhancement (α = 0.84/0.78), exploitativeness (α = 0.72/0.69), grandiose fantasy (α = 0.86/0.74), entitlement rage (α = 0.87/0.75), devaluing (α = 0.86/0.74), grandiosity (α = 0.92/0.88) and vulnerability (α = 0.96/0.91).

### Correlations of the PNI scores with other continuous variables

Table 4 presents the correlations between PNI total scores and external variables and Table 5 (Model 1) presents bivariate associations between PNI Grandiosity/Vulnerability and external variables. The total PNI score was significantly positively associated with negative affect (r = .44), detachment (r = .22), antagonism (r = .21), disinhibition (r = .24), psychoticism (r = .34), depression (r = .30), and neuroticism (r = .30), and significantly negatively associated with self-esteem (r=-.11), premeditation (r=-.18), perseverance (r=-.16), and sensation seeking (r=-.14). Grandiosity was significantly positively associated with negative affect (r = .36), detachment (r = .11), antagonism (r = .11), disinhibition (r = .17), psychoticism (r = .29), depression (r = .20), agreeableness (r = .16), conscientiousness (r = .14), negative emotionality (r = .20) and openness (r = .16). Vulnerability was significantly positively associated with negative affect (r = .45), detachment (r = .27), antagonism (r = .25), disinhibition (r = .26), psychoticism (r = .35), depression (r = .34) and neuroticism (r = .34), and significantly negatively associated with self-esteem (r=-.18), premeditation (r=-.22), perseverance (r=-.21), sensation seeking (r=-.20), and extraversion (r=-.17). Table 6 summarizes the correlations of the B-PNI total score with the other measures.

**Table 4:** Correlation of the PNI subscales with other continuous variables

	1	2	3	4	5	6	7	8	9	10	11	12	13	14	15	16	17	18
1. PNI Total	1																	
2. Negative affect	**0.44**	1																
3. Detachment	**0.22**	**0.43**	1															
4. Antagonism	**0.21**	**0.27**	**0.53**	1														
5. Disinhibition	**0.24**	**0.47**	**0.49**	**0.55**	1													
6. Psychoticism	**0.34**	**0.58**	**0.61**	**0.46**	**0.56**	1												
7. Depression	**0.30**	**0.60**	**0.48**	**0.38**	**0.44**	**0.54**	1											
8. Self-esteem	**− 0.11**	**− 0.14**	**− 0.31**	**− 0.24**	**− 0.24**	**− 0.17**	**− 0.38**	1										
9. Urgency	0.02	**0.13**	**0.14**	**0.22**	**0.31**	**0.19**	**0.16**	**− 0.26**	1									
10. Premeditation	**− 0.18**	**− 0.18**	**− 0.17**	**− 0.25**	**− 0.42**	**− 0.18**	**− 0.24**	**0.30**	− 0.09	1								
11. Perseverance	**− 0.16**	**− 0.16**	**− 0.21**	**− 0.18**	**− 0.23**	**− 0.13**	**− 0.24**	**0.22**	0.10	**0.55**	1							
12. Sensation seeking	**− 0.14**	**− 0.11**	**− 0.28**	**− 0.19**	**− 0.20**	**− 0.17**	**− 0.20**	**0.35**	**0.19**	**0.46**	**0.58**	1						
13. Age	− 0.08	**− 0.14**	− 0.10	− 0.001	**− 0.10**	**− 0.20**	− 0.05	**− 0.10**	0.001	0.06	**0.10**	− 0.01	1					
14. Extraversion	− 0.08	**− 0.16**	**− 0.43**	**− 0.23**	**− 0.12**	**− 0.26**	**− 0.28**	**0.47**	**− 0.15**	**− 0.23**	**0.27**	**0.34**	− 0.02	1				
15. Agreeableness	0.03	0.07	**− 0.36**	**− 0.48**	**− 0.22**	**− 0.20**	**− 0.18**	**0.40**	**− 0.35**	**0.16**	**0.12**	**0.17**	0.04	**0.47**	1			
16. Conscientiousness	0.01	− 0.02	**− 0.32**	**− 0.35**	**− 0.33**	**− 0.23**	**− 0.21**	**0.50**	**− 0.32**	**0.39**	**0.32**	**0.34**	0.06	**0.44**	**0.61**	1		
17. Neuroticism	**0.30**	**0.50**	**0.21**	− 0.01	**0.12**	**0.25**	**0.45**	**− 0.32**	**0.18**	**− 0.17**	**− 0.10**	− 0.07	− 0.07	**− 0.28**	0.003	− 0.06	1	
18. Openness	0.03	0.04	**− 0.21**	**− 0.29**	**− 0.25**	− 0.05	− 0.05	**0.35**	**− 0.26**	**0.29**	**0.25**	**0.27**	**− 0.11**	**0.38**	**0.48**	**0.52**	0.06	1

Numbers in bold indicate significant p-values

**Table 5:** Univariate (Model 1) and Multivariate (Model 2) Regressions with PNI grandiosity and vulnerability predicting each of the personality traits and maladaptive personality traits

	Model 1			Model 2		
	β	CI	R^2^	β	CI	ΔR^2^
Extraversion						
**Grandiosity**	0.10	− 0.003; 0.19	0.009	0.79***	0.63; 0.95	0.213
**Vulnerability**	− 0.17**	− 0.26; − 0.07	0.028	− 0.83***	− 0.99; − 0.67	
**Conscientiousness**						
**Grandiosity**	0.14**	0.04; 0.24	0.019	0.62***	0.45; 0.79	0.115
**Vulnerability**	− 0.05	− 0.15; 0.05	0.003	− 0.57***	− 0.74; − 0.40	
**Agreeableness**						
**Grandiosity**	0.16**	0.06; 0.26	0.026	0.64***	0.47; 0.81	0.123
**Vulnerability**	− 0.04	− 0.13; 0.06	0.001	− 0.57***	− 0.74; − 0.40	
**Neuroticism**						
**Grandiosity**	0.20***	0.10; 0.30	0.040	− 0.29**	− 0.45; − 0.12	0.139
**Vulnerability**	0.34***	0.25; 0.43	0.115	0.58***	0.41; 0.75	
**Openness**						
**Grandiosity**	0.16**	0.06; 0.26	0.025	0.65***	0.48; 0.82	0.126
**Vulnerability**	− 0.04	− 0.14; 0.06	0.002	− 0.58***	− 0.75; − 0.41	
**Negative affect**						
**Grandiosity**	0.36***	0.27; 0.45	0.131	− 0.04	− 0.20; 0.12	0.201
**Vulnerability**	0.45***	0.36; 0.54	0.200	0.48***	0.32; 0.65	
**Detachment**						
**Grandiosity**	0.11*	0.01; 0.21	0.012	− 0.37***	− 0.54; − 0.20	0.111
**Vulnerability**	0.27***	0.17; 0.36	0.070	0.58***	0.41; 0.75	
**Antagonism**						
**Grandiosity**	0.11*	0.01; 0.20	0.011	− 0.36***	− 0.53; − 0.19	0.102
**Vulnerability**	0.25***	0.16; 0.35	0.064	0.55***	0.38; 0.72	
**Disinhibition**						
**Grandiosity**	0.17**	0.07; 0.26	0.028	− 0.19*	− 0.36; − 0.01	0.080
**Vulnerability**	0.26***	0.17; 0.36	0.070	0.42***	0.25; 0.59	
**Psychoticism**						
**Grandiosity**	0.29***	0.19; 0.38	0.083	− 0.01	− 0.18; 0.17	0.120
**Vulnerability**	0.35***	0.25; 0.44	0.119	0.35***	0.18; 0.52	
**Depression**						
**Grandiosity**	0.20***	0.10; 0.29	0.039	− 0.29**	− 0.46; − 0.13	0.140
**Vulnerability**	0.34***	0.25; 0.43	0.115	0.58***	0.42; 0.75	
**Self-esteem**						
**Grandiosity**	0.04	− 0.06; 0.14	0.002	0.65***	0.48; 0.81	0.156
**Vulnerability**	− 0.18***	− 0.28; − 0.08	0.032	− 0.72***	− 0.89; − 0.56	
**Urgency**						
**Grandiosity**	− 0.06	− 0.16; 0.04	0.004	− 0.37***	− 0.54; − 0.19	0.043
**Vulnerability**	0.06	− 0.04; 0.16	0.003	0.37***	0.19; 0.54	
**Premeditation**						
**Grandiosity**	− 0.07	− 0.17; 0.03	0.005	0.39***	0.22; 0.56	0.095
**Vulnerability**	− 0.22***	− 0.32; − 0.13	0.049	− 0.55***	− 0.72; − 0.38	
**Perseverance**						
**Grandiosity**	− 0.06	− 0.16; 0.04	0.003	0.39***	0.22; 0.56	0.087
**Vulnerability**	− 0.21***	− 0.30; − 0.11	0.042	− 0.53***	− 0.70; − 0.36	
**Sensation seeking**						
**Grandiosity**	− 0.02	− 0.12; 0.08	< 0.001	0.50***	0.33; 0.67	0.114
**Vulnerability**	− 0.20***	− 0.30; − 0.10	0.039	− 0.62***	− 0.79; − 0.45	

Model 1: Grandiosity and vulnerability entered separately in each regression analysis; Model 2: both variables entered together in the model. *p < .05; **p < .01; ***p < .001

**Table 6:** Correlation of the B-PNI subscales with other continuous variables

	1	2	3	4	5	6	7	8	9	10	11	12	13	14	15	16	17	18
1. B-PNI Total	1																	
2. Negative affect	**0.45**	1																
3. Detachment	**0.20**	**0.43**	1															
4. Antagonism	**0.14**	**0.28**	**0.53**	1														
5. Disinhibition	**0.23**	**0.47**	**0.49**	**0.55**	1													
6. Psychoticism	**0.31**	**0.58**	**0.61**	**0.46**	**0.56**	1												
7. Depression	**0.28**	**0.60**	**0.48**	**0.38**	**0.44**	**0.54**	1											
8. Self-esteem	− 0.07	**− 0.14**	**− 0.31**	**− 0.24**	**− 0.24**	**− 0.17**	**− 0.38**	1										
9. Urgency	− 0.01	**0.13**	**0.14**	**0.22**	**0.31**	**0.19**	**0.16**	**− 0.26**	1									
10. Premeditation	**− 0.16**	**− 0.18**	**− 0.17**	**− 0.25**	**− 0.42**	**− 0.18**	**− 0.24**	**0.30**	− 0.09	1								
11. Perseverance	**− 0.15**	**− 0.16**	**− 0.21**	**− 0.18**	**− 0.23**	**− 0.13**	**− 0.24**	**0.22**	0.10	**0.55**	1							
12. Sensation seeking	**− 0.13**	**− 0.11**	**− 0.28**	**− 0.19**	**− 0.20**	**− 0.17**	**− 0.20**	**0.35**	**0.19**	**0.46**	**0.58**	1						
13. Age	− 0.06	**− 0.14**	− 0.10	− 0.001	**− 0.10**	**− 0.20**	− 0.05	**− 0.10**	0.001	0.06	**0.10**	− 0.01	1					
14. Extraversion	− 0.04	**− 0.16**	**− 0.43**	**− 0.23**	**− 0.12**	**− 0.26**	**− 0.28**	**0.47**	**− 0.15**	**0.23**	**0.27**	**0.34**	− 0.02	1				
15. Agreeableness	**0.10**	0.07	**− 0.36**	**− 0.48**	**− 0.22**	**− 0.20**	**− 0.18**	**0.40**	**− 0.35**	**0.16**	**0.12**	**0.17**	0.04	**0.47**	1			
16. Conscientiousness	0.06	− 0.02	**− 0.32**	**0.35**	**− 0.33**	**− 0.23**	**− 0.21**	**0.50**	**− 0.32**	**0.39**	**0.32**	**0.34**	0.06	**0.44**	**0.61**	1		
17. Neuroticism	**0.31**	**0.50**	**0.21**	− 0.01	**0.12**	**0.25**	**0.45**	**− 0.32**	**0.18**	**− 0.17**	**− 0.10**	− 0.07	− 0.07	**− 0.28**	0.003	− 0.06	1	
18. Openness	**0.10**	0.04	**− 0.21**	**− 0.29**	**− 0.25**	− 0.05	− 0.05	**0.35**	**− 0.26**	**0.29**	**0.25**	**0.27**	**− 0.11**	**− 0.38**	**0.48**	**0.52**	0.06	1

Numbers in bold indicate significant p-values

### Regression analyses

Regression analyses are presented in Table 5. In the recommended multivariate regressions (Model 2), grandiosity and vulnerability exhibit more distinctive associations consistent with the assessed constructs, replicating Edershile and colleagues [48]. Specifically, grandiosity was significantly positively associated with extraversion, conscientiousness, agreeableness, openness, self-esteem, premeditation, perseverance, and sensation seeking, and significantly negatively associated with neuroticism, depression, urgency, detachment, antagonism, and disinhibition. Vulnerability was significantly positively associated with neuroticism, negative affect, detachment, disinhibition, antagonism, psychoticism, depression, and urgency, and significantly negatively associated with extraversion, conscientiousness, agreeableness, openness, self-esteem, premeditation, perseverance, and sensation seeking.

### Gender differences

A lower mean exploitativeness score was found in females compared to males (17.49 vs. 15.83; p = .004). No significant difference was observed by gender for the other scores (Table 7).

**Table 7:** Comparison of PNI scores between sexes

	Males	Females	t	*p*	Cohen’s d
1. PNI Contingent self-esteem	2.86 ± 1.19	2.76 ± 1.19	0.789	0.430	0.087
2. PNI exploitativeness	3.50 ± 1.02	3.17 ± 1.04	2.885	**0.004**	0.322
3. PNI self-sacrifice self-enhancement	3.39 ± 1.14	3.35 ± 1.21	0.286	0.775	0.032
4. PNI hiding the self	3.58 ± 1.08	3.48 ± 1.21	0.813	0.417	0.092
5. PNI grandiose fantasy	3.50 ± 1.08	3.50 ± 1.23	− 0.061	0.951	0.006
6. PNI lack of self-esteem	3.14 ± 1.11	3.12 ± 1.22	0.131	0.896	0.014
7. PNI entitlement rage	3.23 ± 1.05	3.19 ± 1.20	0.320	0.750	0.034
8. PNI Grandiosity	3.46 ± 0.94	3.36 ± 1.05	0.939	0.349	0.100
9. PNI Vulnerability	3.15 ± 0.98	3.08 ± 1.08	0.637	0.525	0.068
10. PNI total score	3.26 ± 0.93	3.18 ± 1.03	0.769	0.443	0.081

## Discussion

During the last decade, research and clinical interest in narcissism has risen [2], and one study found Arab females scored higher in narcissistic traits than European counterparts [33], possibly due to cultural differences. Narcissism may result in debilitating consequences [7, 18, 20, 49], thus the importance of its assessment, monitoring and management. However, this could not be possible without valid and reliable measures. The present work was motivated by the fact that no valid narcissism measure is available so far in the Arabic language. We thus aimed to examine the factor structure and psychometric properties of the Arabic PNI and B-PNI. Our results confirmed the seven-factor structure with reliable subscales and two intercorrelated higher-order factors of vulnerability and grandiosity for both the Arabic PNI and Arabic B-PNI. As expected, when considered from a multivariate perspective, vulnerability and grandiosity demonstrated distinctive patterns of relationships with normal and maladaptive personality traits, depression, self-esteem, and impulsivity, thus supporting the discriminant validity of grandiosity and vulnerability. These results suggest that scores on the full-length and brief-form Arabic versions of the PNI are reliable and valid measures of pathological narcissism and can be used to assess this construct in Arabic-speaking populations.

In terms of factorial structure, both versions of the scale replicated the previously hypothesized seven first-order factors of the original versions [7, 9], as well as other subsequent validation studies, thus confirming the multidimensionality of the PNI. The seven aspects of PNI are contingent self-esteem, grandiose fantasy, self-sacrificing self-enhancement, devaluing, exploitativeness, hiding the self, and entitlement rage. CFA supported a hierarchical structure with seven-factor lower-order and two intercorrelated higher-order factors for the Arabic translation, ensuring factor structures consistent with the original and brief PNI. The two-factor higher order solution for the PNI supported in the study are grandiose fantasy, self-sacrificing self-enhancement, and exploitativeness representing Narcissistic Grandiosity and entitlement rage, contingent self-esteem, devaluing, and hiding the self-representing Narcissistic Vulnerability. The results showed that the standardized factor loading of EXP on Narcissistic Grandiosity in this study was higher than the values reported in another study [29] but lower than the value reported by Wright et al. [8]. This can be explained by the fact that EXP is not a strong marker of either Pathological Narcissism or Narcissistic Grandiosity, which was confirmed by another previous study [50]. The controversial findings associated with EXP can be explained by the issues associated with measurement and construct definition where EXP mainly evaluates thoughts and behaviors oriented towards other people, while SSSE and GF scales help detect more internally focused aspects of self-concept associated with pathological narcissism.

Cronbach’s alpha values ranged from 0.72 to 0.93 for the Arabic PNI, and from 0.59 to 0.81 for the Arabic B-PNI, thus attesting the adequacy of internal consistency coefficients of these scales. Similar reliability estimates have been documented in other previous validation studies [29, 32]. For instance, the two second- order factors showed high internal consistencies and further confirm the second-order structure previously obtained among Croatian, American, and Chinese university students [8, 29, 32]. More specifically, our study showed that grandiose fantasy, exploitativeness, and self-sacrificing self-enhancement loaded on Narcissistic Grandiosity, whereas contingent self-esteem, devaluing, entitlement and hiding the self-loaded on Narcissistic Vulnerability. While measurement invariance across genders could not be performed in the current study, the comparison of PNI scores between male and female participants found no differences, except for the exploitativeness dimension, which was higher in males. These findings are consistent with the findings from prior research [8, 29], and can be explained by the positive correlation of exploitativeness with interpersonal dominance, a style that tend to be more associated with males [51].

When adjusting for the overlap between PNI Vulnerability and PNI Grandiosity using multivariate regression analyses as recommended by Edershile et al. [48], the results showed two distinct patterns of associations with personality traits. Grandiosity was linked to more adaptive traits (i.e., grandiosity, conscientiousness, agreeableness, openness) [52, 53], whereas vulnerability was rather positively related to maladaptive traits (i.e., neuroticism, negative affect, detachment, and psychoticism) [54]. While the majority of our findings are in agreement with those of prior studies, one unusual finding stands out. For the Arabic PNI, when controlling for vulnerability, grandiosity is positively associated with agreeableness and negatively associated with antagonism. In prior studies, both grandiosity and vulnerablility are negatively associated with agreeableness and positively linked to antagonism [48]. Here, this pattern only emerges for vulnerability. Additional research is required to determine if this is a nomological association unique to Arabic culture. Overall, our results suggest that the Arabic PNI and B-PNI adequately represented the narcissistic grandiosity dimension as typically manifested by an exaggerated sense of self-importance [55], but perhaps in a less interpersonally hostile way. Also, both the PNI and the B-PNI appropriately capture and differentiate between the grandiose and vulnerable facets of pathological narcissism.

### Limitations

Some limitations of this study could be improved upon in future research. First, information was collected among young adults using social media, with the majority being females, single and having a high level of education, which leads to a lack of representativeness of the sample. Second, the data collected only relied on self-report evaluation that might be associated with social desirability and self-deceptive responsiveness, both being extremely important in the case of narcissism. However, prior research with the PNI suggested self- and informant-reports converge [56]. Further studies are highly recommended to apply a multi-method strategy for data collection (e.g., interviews, informant reports). Furthermore, the present study failed to evaluate some psychometric characteristics of the PNI and B-PNI (such as test-retest reliability and invariance across gender). Finally, the Arabic version has been validated in a non-clinical adult sample, precluding generalization to clinical populations. Narcissistic pathology exists on a continuum of severity from normal to pathological within the general population. Both the original PNI [7] and several previous translations of the PNI (e.g., [12, 13, 57]) were constructed using university student and community dwelling adult samples. However, future validation studies still need to confirm the robustness of the Arabic PNI psychometric qualities in clinical settings.

## Conclusion

The present results suggest the Arabic PNI is a reliable research instrument of broadly defined pathological narcissism. The scale demonstrated satisfactory structural and convergent validity among a Lebanese community young adult population. The scale was translated in the official written Arabic language; therefore, it can be used in other Arabic-speaking countries. Given the well-established high clinical relevance of pathological narcissism, we hope that making the valid, reliable and widely used self-report PNI available in the Arabic language will extend its use for clinical and research purposes among the broader Arabic-speaking population around the globe. We also offer to Arab clinicians and researchers the B-PNI, a briefer and more convenient form of the scale that will hopefully encourage more cross-cultural research on pathological narcissism.

## Data Availability

All data generated or analyzed during this study are not publicly available due to restrictions imposed by the ethics committee. The dataset supporting the conclusions is available upon request to the corresponding author (SH).
